# Updated Organic Composition and Potential Therapeutic Properties of Different Varieties of Olive Leaves from *Olea europaea*

**DOI:** 10.3390/plants12030688

**Published:** 2023-02-03

**Authors:** Diana Melo Ferreira, Natália M. de Oliveira, Maria Helena Chéu, Diana Meireles, Lara Lopes, Maria Beatriz Oliveira, Jorge Machado

**Affiliations:** 1 LAQV/REQUIMTE—Department of Chemical Sciences, Faculty of Pharmacy, University of Porto, 4050-313 Porto, Portugal; 2 Laboratory of Applied Physiology, Institute of Biomedical Sciences Abel Salazar—ICBAS, University of Porto, 4050-313 Porto, Portugal; 3 Centre of Biosciences in Integrative Health—CBSin, 4250-105 Porto, Portugal; 4 RECI—Research Unit in Education and Community Intervention, Instituto Piaget—ISEIT, 3515-776 Viseu, Portugal

**Keywords:** *Olea europaea* L. *folium*, phenolic compounds, vitamin E, fatty acids, nutraceutical potential, intelligent bioactive compounds

## Abstract

*Olea europaea* L. *folium* merits further exploration of the potential of its substrates for therapeutic supplements. Quantitative and qualitative analyses were conducted on samples of Madural, Verdeal, and Cobrançosa elementary leaves and leaf sprouts (*mamões*) collected in the region of Valpaços, Portugal. Organic analysis assessed the moisture content, total carbohydrates, ash, protein, and fat contents, total phenolic content (TPC), vitamin E, and fatty acid (FA) profiles. Moisture content was determined through infrared hygrometry and TPC was determined by a spectrophotometric method. Concerning organic analysis, all leaf samples showed similar moisture content, though Cobrançosa’s leaf sprouts and Verdeal’s elementary leaves had slightly lower contents. Meanwhile, these cultivars also showed a higher TPC, α-tocopherol isomer, and fatty acid composition (FAC). FAC in all samples exhibited higher contents of PUFA and SFA than MUFA, with a predominance of linolenic and palmitic acids. Organic analyses of Cobrançosa’s leaf sprouts and Verdeal’s elementary leaf extracts allow for the prediction of adequate physiological properties regarding neuroinflammatory, neurobehavioral, metabolic, cardiovascular, osteo-degenerative, anti-ageing, pulmonary, and immunological defense disorders. These physiological changes observed in our preliminary in silico studies suggest an excellent nutraceutical, which should be borne in mind during severe pandemic situations.

## 1. Introduction

*O. europaea* L., typically referred to as the olive tree, has been cultivated in the Mediterranean land since prehistoric times. The olive fruit, its oil, and the tree leaves have a rich history of nutritional and medicinal value. This fruity oil was used for several purposes, from cooking and fueling lamps to ceremonial rites, traditional medicine, and cosmetics [1]. Today’s olive production is mostly exploited for the generation of this golden oil and the main actors responsible for the major volume of olive oil are still within Europe (Spain > Greece > Italy > Portugal). So, olive production has a significant impact on the agricultural economy of Mediterranean countries such as Spain, Greece, Italy, and Portugal. The characteristics and properties of *O. europaea* L. are naturally determined by the climate, soil, cultivar, ripening stage of the fruit, processing period after harvest, etc. [2]. In the region of Trás-os-Montes in northern Portugal, where we harvested the samples for our work, numerous cultivars of olive trees appeared, showing the variability of morphological characteristics, and expressing distinct qualities and great commercial value in their products. However, they are confined to altitudes <600–700 m denominated “Terra Quente” (Warm Land) and to average temperatures above 12.5 °C, consequently disappearing in altitudes >800 m, Terra Fria (Cold Land) [3]. As mentioned, olive culture in the Trás-os-Montes region is practiced primarily in Transmontane Warm Land, where the municipalities of Mirandela, Macedo de Cavaleiros, Valpaços, and Vila Flor stand out, as well as Alfândega da Fé, Torre de Moncorvo, Mogadouro, Vila Nova de Foz Côa, among others as Freixo de Espada à Cinta for olive-canning, which together represent about 75% of the regional olive grove area and 98% of the table olive production. In Trás-os-Montes, an estimate from the year 2005 and for a total of about 11.5 million olive trees, the following varieties were considered in order of importance as a percentage of the number of olive trees: Cobrançosa (30%), Verdeal Transmontana (24%), Madural (19%), Cordovil (10%), Negrinha de Freixo (5%), Santulhana (4%), and others (8%) [4]. According to the information of the local people, given that the increase of new areas and the reconversion of old olive groves (with little significance) were mainly based on the varieties Cobrançosa (85 to 90%), Madural (5 to 6%), Negrinha de Freixo (2 to 3%), and others (<2%), the changing perspective will only have undergone a slight adjustment. Another fact of relevant importance is the continued dominance of the Cobrançosa variety in the new olive groves for olive oil production, accounting for more than 85% because it is a variety amenable to easy multiplication by herbaceous cutting, very abundant and productive in medium fertile soils, suitable for mechanical harvesting, and of good olive oil yield [4]. In this area, variable choices should be guided not only by technical criteria such as the adaptation of varieties to local micro-climatic conditions, rusticity, vigor, and size of the tree, production, and mixing of varieties, but also by criteria of economic sustainability; a type of production for desired markets and varieties that correspond well to new technologies for controlling production factors. According to *Instituto Nacional de Estatística* (INE), in 2021, the sum of these parcels to others in Trás-os-Montes’ occupied a total area of the olive grove of about 81,000 ha (21.7%), involving 37,000 olive growers (INE, 2021- https://www.acos.pt › files › 20210401145737753recenseamento-agricola-2019-2021.pdf, accessed on 5 January 2023), with a production of around twenty million liters of olive oil (INE, 2022, https://www.ine.pt/xportal/xmain?xpid=INE&xpgid=ine_indicadores&indOcorrCod=0000709&contexto=bd&selTab=tab2&xlang=PT, last accessed on 5 January 2023). Among the most expressive cultivars, some originate an olive oil of exceptional quality, which is the reason for the existence of the Protected Designation of Origin (DOP) by the European Union, since 1996, for olive oil of Trás-os-Montes. The relevance of *O. europaea* L. cultivation has always been the production of olive oil, which still holds indispensable value in our daily diet alongside other social, cultural, and economic benefits [5]. Extraction of other by-products, such as olive leaves (*O. europaea* L. *folium*), might serve diverse purposes including the therapeutic application as a complementary and medicinal product that will be highlighted in this work. Both products are of fundamental importance in human nutrition and diet, being notably valued for their biological properties and organoleptic characteristics [6]. Olive leaves are one of the main by-products and, overall, they contribute about 10% of the weight of olives harvested for oil extraction, bearing in mind that volumes may vary depending on culture conditions, tree age, production, and/or local pruning practices [7,8]. Usually, the olive mill leaves become a mammoth of unusable material taken for animal feed but mostly ending up being burned, ground, and disposed in landfills [9]. This treatment as a waste by-product potentially brings economic and environmental issues beyond from the producer’s removal, storage, and elimination costs [10]. Nevertheless, the olive leaf could be used to obtain value-added products, given the kind of bioactive compounds that have been extracted from them and further studied. Regarding its use in traditional herbal medicine, particularly in the Mediterranean region, olive leaves have extensive application with the aim of preventing and treating several diseases, namely, metabolic alterations such as diabetes, cardiovascular disease (CVD), cancer, neurogenerative ailments, and another number of health problems [11,12,13,14,15,16,17,18]. The olive tree has been widely accepted as one of the species with the highest antioxidant activity via the phenolic components in its oil, fruits, and leaves [19,20]. Phenolic compounds found in the olive leaf can belong to different classes: phenolic acids, such as caffeic, gallic, vanillic and coumaric acids; phenolic alcohols including tyrosol (TY) and hydroxytyrosol (HT); and more complex compounds such as secoiridoids (oleuropein (OLEP), ligstroside), lignans (acetoxypinoresinol and pinoresinol), flavonoids, and finally hydroxyl-isochrons [21,22,23,24]. The main active phenolic compound in olive leaf is OLEP, a phenylethanoid found in olive oil and leaves together with other closely related compounds—10-hydroxyoleuropein, ligstroside, and 10-hydroxyligstroside—to which attributed nutraceutical properties are attributed [25,26]. Among the polyphenols identified in olive leaf extract (OLE), both OLEP and HT are considered the major candidates for pharmacological use, both as a single drug or after the enrichment of olive oil or other food components. Findings obtained with oleuropein aglycone or some semisynthetic suggest that it is possible to improve the pharmacological properties of the compounds and consequently offer their wide utilization in human pharmacology [27]. Moreover, fatty acids such as oleic acid (OA) from part of the main bioactive component identified in OLE. OA has attracted much attention as a protective agent against tumoral cells in both epidemiological and animal studies [28,29,30,31,32]; diet–drug combinations of chemotherapy with nutraceuticals might have the potential to deliver synergistic protection against the progression of cancer [33]; and, last but not the least, OLE was used in some countries where SARS-CoV-2 infection was widespread [34,35]; this application was supported by the antioxidant [36,37], cardioprotective [38], and anti-inflammatory [39] biological activities.

Thus, this work aimed to update the organic composition of different varieties of olive leaves (including elementary leaves and leaf sprouts) and highlight their potential therapeutic properties based on the obtained results and the available literature.

## 2. Results

### 2.1. Organic Profile

The nutritional analysis of the different cultivars’ leaves demonstrated, for Madural and Cobrançosa’s leaves, a predominance of: *Total carbohydrates > Moisture > Total protein > Ash > Total fat*. As for the remnant samples, the predominance was as follows: *Total carbohydrates> Total protein > Moisture > Ash > Total fat.* Total carbohydrates were the highest content of all nutrients studied, and its predominance among elementary leaves were as follows: Madural > Cobrançosa > Verdeal; for leaf sprouts: Madural > Verdeal > Cobrançosa. Moisture analysis showed similar results among the three studied varieties with a slightly lower value for Cobrançosa’s leaf sprouts, 8.43 ± 0.11, and Verdeal leaves, 8.61 ± 0.12, respectively (Table 1). Total protein content was higher for leaf sprouts in all cultivars. The highest total protein content distributed among cultivars was as follows: Cobrançosa > Verdeal > Madural. Contrary to the protein content, the total amount of fat found in the elementary leaf samples was higher compared to leaf sprouts except in Cobrançosa’s variety. The analysis for the total fat found an almost doubled value for the Verdeal cultivar’s leaf—*circa* 4.01% (Table 1) compared to the relative 2.15% found in Cobrançosa’s. Meanwhile, the leaf sprouts of both cultivars were showed to have a similar percentage of total fat of 2.45% (Verdeal) and 2.38% (Cobrançosa) (Table 1), almost doubling the 1.77% of relative fat found in Madural’s (Table 1). Percentage of ash was higher in elementary leaves, except in Madural’s cultivar, where leaf sprouts scored a higher percentage. Ash percentage varied in elementary leaves: Cobrançosa > Verdeal > Madural; for leaf sprouts: Madural > Cobrançosa > Verdeal.

In general, the leaf sprouts of all the three cultivars were shown to be richer in total phenolic content (TPC) compared to their respective leaf sample (Table 2). Cobrançosa’s leaves and leaf sprouts showed a similar value—3.37 g GAE/100 g and 3.78 g GAE/100 g, respectively (Table 2)—but was slightly higher compared to the other two varieties.

The vitamin E profile for both elementary leaves and leaf sprouts showed α-tocopherol (α-T) > γ-tocopherol (γ-T) > β-tocopherol (β-T) > δ-tocopherol (δ-T). Total tocopherols were found in much higher contents in elementary leaves for all cultivars. Leaves of the Verdeal variety showed a higher total of tocopherols—21.61 mg/100 g of leaves in fresh weight (Table 3). This almost doubled the tocopherols in Madural’s leaves—12.03 mg/100 g (Table 3). Considering both total tocopherol and α-T, it is possible to identify that its content in elementary leaves and leaf sprouts decreases in as follows: Verdeal > Cobrançosa > Madural. β-T was demonstrated to be the lesser value of all the isomers detected in leaves and sprout leaves of all varieties, except for the Verdeal’s elementary leaves, where β-T and γ-T showed an equal value of 0.32 mg/100 g (Table 3). When studying the tocopherol profile for elementary leaves, it was possible to identify α-T as the major relative E vitamer: 97.04% (Verdeal) > 96.01% (Madural) > 95.37% (Cobrançosa) (Table 3).

Fatty acids’ (FA) profile for both elementary leaves and leaf sprouts identifies a relative major presence of PUFA > SFA > MUFA for all cultivars, except for the Cobrançosa’s leaf sprouts, where SFA > PUFA > MUFA. Linolenic acid (ALA, C18:3n3) was identified as the most predominant of all PUFAs for elementary leaves and leaf sprouts of all cultivars: Verdeal > Madural > Cobrançosa (Table 4). Compared to ALA, omega-6 linoleic acid (LA, C18:2n6c) comes second, showing a relative presence of about half of ALA values for all the samples, regardless the cultivar.

Total content of MUFA appears as the lesser value compared to total PUFA and SFA for all samples of each cultivar. Among MUFA, a relative higher content of oleic acid (OA, C18:1n9c) was determined compared to a vestigial presence of cis-11-eicosanoic acid (C20:1n9) for all samples. OA values in leaf sprouts are slightly higher than elementary leaves for all the cultivars, with an advantage for Cobrançosa’s samples where OA values found a total of 16.24% and 20.66% (Table 4), respectively.

Total SFA comes second in the FA profile for all cultivars, except for Cobrançosa’s leaf sprouts. These showed SFA content slightly higher than PUFA. The SFA relative profile followed: C16:0 > C18:0 > C14:0 > C20:0. Palmitic acid (PA, C16:0) was the relative major SFA component with very approximate values for elementary leaves of all cultivars.

### 2.2. In Silico Integrator: Tests of Substance Effects

In hypertension or immunological depressed patients, *O. europaea* was tested as a positive potential on the homeostasis balance for the nervous and circulatory systems, cognitive and hormonal functions, and the heart activity. Although the potential effect on the small intestine and their general immune system functions were not significant for this clinical complexion, these activities were not negatively disturbed; instead, they may support natural defenses.

In oncological patients, the circulatory system and the connective tissue register potential improvements. On the other hand, we do not obtain a positive impact for the lymphatic degenerative tendency and immunological system, but they can, in any case, also contribute to homeostasis maintenance. Finally, for neurodevelopment disorders (children with Autism), we recorded a positive impact in cognitive functions with no significant difference for the immunological functions (though it helps to maintain them when healthy).

## 3. Discussion

### 3.1. Nutritional Composition

Despite most of the samples have shown a predominance of *Total carbohydrates> Total protein > Moisture > Ash > Total fat*, the elementary leaves of Cobrançosa and Madural, exhibited differently: *Total carbohydrates > Moisture> Total protein > Ash > Total fat.* In the recent literature, olive leaves proved valuable for developing functional dietetic goods and health enhancement for which the harvest and conservation of both leaves and leaf sprouts should be addressed properly, guaranteeing quality. Their prompt drying in the post-harvesting period avoids compromising their quality and possible degeneration during storage; leaves are often dried before any extraction to reduce their total moisture, hence preventing the interference of their water content in the polyphenol leaching on extract composition and antioxidant potential [40]. Eliminating the moisture potentiates the phenolic compounds and their precious antioxidant activity within the extracts of both elementary leaves and leaf spouts of *O. europaea* [41,42,43]. Although air drying at room temperature has been a traditional method good enough to protect bioactive compounds from deterioration, hot air drying is mostly used at the industrial scale as it shortens the process and allows several parameters to be controlled so that the dried products can achieve the highest quality, even when compared to vacuum freeze drying. Ahmad-Quasem et al., in 2012, revealed that major polyphenols such as OLEP, verbascoside, and luteolin-7-O-glucoside, identified in extracts, had been influenced by both leaf freezing and drying techniques, regardless of the freezing technique. Freeze dried leaves did not gather extracts with a high antioxidant potential; hot air drying of fresh leaves at high temperatures (120 °C) is still considered the most suitable method to strengthen the antioxidant potential and OLEP content of extracts for industrial purposes [40]. In that sense it is safe to say that Cobrançosa’s leaf sprouts and Verdeal’s elementary leaves (Table 1), for their relatively lower moisture values of, could be the most useful substrates for retaining a high-quality phenolic composition.

Regarding protein content, the highest value belongs to Cobrançosas’s leaf sprouts, which also have the least of amount of water of all samples. These seem to be a suitable substrate for potentiating rich polyphenol extraction, protected from the proteolytic activity associated with the degradation of leaf when exposed to sun and other conditions.

Elementary leaves of the Verdeal cultivar, with 4.01% of total fat content (Table 1), also showed the highest percentage of omega-3 PUFA (linolenic acid) (Table 4), contrary to the elementary leaf of Cobrançosa which showed the highest values for MUFA content, namely, of omega-9 FA (oleic acid) (Table 4). Considering that PUFAs are considered essential FA for the human body, the Verdeal elementary leaf provides a more valuable subtrate compared to Cobrançosa, as MUFA are considered only partially essential FA. On the other hand, carbohydrate content was identified to be more present in the Madural cultivar opposite to the Cobrançosa and Verdeal; it was shown to have a relatively higher presence of proteins. It is now known that a high-carbohydrate meal also promotes the inflammatory activation of macrophages, major players implicated in the inflammatory response observed in the obese adipose tissue, the same way high-fat diet-induced obesity is linked to a chronic state of low-grade inflammation with higher risk of T2DM related to obesity, CVD, musculoskeletal disorders, and cancer [44]. So, nutritional results suggest that Cobrançosa and Verdeal’s leaf sprouts (though close to respective elementary leaves values—Table 1) are healthier options to be used as nutraceutical source.

### 3.2. Total Phenolic Composition

**Figure 1:**
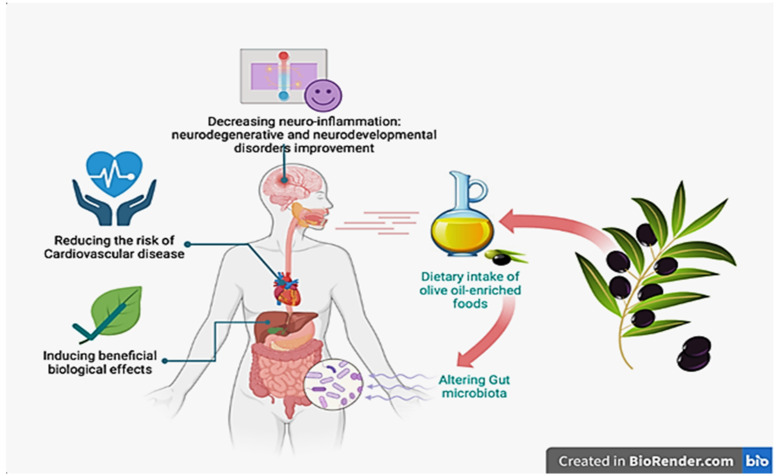
Illustration adapted with permission from Ref [81], 2019 Gavahian M. et al., under the Creative Commons License (License 5480141442531). Adapted by https://Biorender.com (2023) and https://Qvectors.net (accessed on 5 January 2023), vector database for free use.

Olive tree polyphenols showed evidence of relieving or even preventing demyelination, neurodegeneration, antihypertensive and cardioprotective effects, while demonstrating anti-inflammation, antioxidant, antimicrobial and immune protective, hypocholesterolemic, hypoglycemic, anti-thrombotic, and anti-cancer properties [32,45]. In this study, the leaf sprouts of all the three cultivars were showed to be richer in total phenol content (TPC) compared to the respective elementary leaf sample (Table 2). Cobrançosa’s leaf sprouts and elementary leaves showed a similar value—3.37 g GAE/100 g and 3.78 g GAE/100 g respectively (Table 2)—slightly higher compared to the other two varieties. Once again, Cobrançosa’s leaf sprouts are shown to be a valuable, cheap, and reliable source of extract polyphenols of high quality; consider the highest value of TPC (Table 2), highest amount of protein (Table 1), and lowest content of water (Table 1). Thus, the TPC in olive leaves, especially Cobrançosa’s leaf sprouts, show a valuable potential use for prevention of risks of CVD (Figure 1) based on the cardioprotective, hypoglycemic, and hypolipidemic properties already addressed in previous studies [46,47,48,49,50,51]. Anti-hypertensive drugs have been a classic treatment for hypertension and subsequent renal and heart ailments. Studies of dual treatment showed some benefits; however, adverse effects of combination therapy bring costs to bear on the health of the treated individuals; for example, hyperkalemia, symptomatic hypotension, and an excess decline in estimated glomerular filtration rate (eGFR) [52] that can be overcome with prophylactic and alternative solutions such as applications of Cobrançosa’s leaf sprouts for their rich TPC and low water content.

According to the World Health Organization (WHO), the number of people with diabetes rose from 108 to 463 million within the last 20 years and the prevalence of hyperglycemia lies between 7% and 14% [53]. Similar to other metabolic diseases, diabetes is a multifaceted disease. Evidence shows insulin resistance (IR) is preceding and central to the development of T2DM, IR being defined physiologically as a state of low response in insulin-targeting tissues to high physiological insulin levels preceding non-physiologic elevated plasma glucose levels, which is the primary clinical symptom of T2DM [54,55,56]. While there are many drugs targeting insulin secretion, only two drugs targeting IR are available [57]. Among anti-diabetic prescribed drugs, glucagon-like peptide 1 (GLP-1) agonists target the ability of β-cells to secrete insulin while thiazolidinediones (TZDs) and metformin are insulin-sensitizing antidiabetic drugs, targeting the fat storage capacity of adipose tissue and glucose production in the liver, respectively. Since GLP-1 has poor stability, GLP-1 agonists with longer half-lives and dipeptidyl peptide-4 (DPP-4) inhibitors are used in combination to treat T2DM. Thiazolidinediones (TZDs) enhance insulin sensitivity in skeletal muscle, liver, and adipose tissue and promote fat redistribution from liver and skeletal muscle to adipocytes [58,59,60]. Metformin, the most commonly prescribed drug for diabetes, may also increase insulin sensitivity in peripheral tissues during fasting [61,62,63,64,65,66]. One of the mechanisms thought to be behind the protective effect of phenolic compounds on the T2DM is that OLE may increase glucagon-like peptide-1 (GLP-1) secretion both in vivo and in vitro [67]. OLE supplementation has been reported to reduce glucose and insulin excursion after oral glucose challenge, suggesting an improvement in both pancreatic β-cell function and insulin sensitivity. Compared to drugs that only improve insulin secretion, OLE of Cobrançosa’s leaf sprouts—which are high in TPC—might improve both insulin sensitivity and pancreatic β-cell secretory capacity, thus resembling metformin therapeutics effects, translating clinical meaning for patients with T2DM. So, Cobrançosa’s leaf sprouts can also be suggested as a premium choice nutraceutical to improve IR in patients developing endocrine disorders (Figure 1). Phenolic compounds are also known for a wide spectrum of antibacterial effects demonstrated to be potent against several strains of bacteria responsible for intestinal and respiratory infections in vitro [29,68,69], showing promise as preservatives for the food industry [70,71,72,73], reducing the growth of *Helicobacter pylori*, an epidemiologic burden of a pathogen associated with peptic ulcer and gastric cancer [68]. Despite works suggesting that olive oil polyphenols exhibit antibacterial activity against the beneficial bacteria *L. acidophilus* and *B. bifidum* [74,75], OLE polyphenols were shown to improve probiotic bacteria growth and metabolism (Figure 1), in particular *B. infantis* and *L. acidophilus*; thus, OLE in the human diet might have the same effect on desirable components of the intestinal microflora [76]. Researchers identify the oxygen scavenger, reducing the redox potential of the phenolic compounds of OLE, as responsible for pH reduction [77,78,79] in the growth media, promoting probiotic bacteria growth in low oxygen ambience [80].

OLE polyphenols have shown antiviral activities [76,77,82,83,84,85,86,87] and antiprotozoal activities, including both antitrypanosomal and antileishmanial activity; thus, it is safe to suggest that Cobrançosa’s leaf sprouts and elementary leaves are both viable sources of TPC to study in the treatment of CVD, IR promoting euglycemia, and hypertension but, also to boost immune action against bacterial, viral, and protozoal infections.

### 3.3. Tocopherol Isomers in O. europaea L. folium

In the present study, the Vitamin E profile showed α-tocopherol (α-T) > γ-tocopherol (γ-T) > β-tocopherol (β-T) > δ-tocopherol (δ-T). α-tocopherol has the ability to (1) pass into biological membranes, preventing protein oxidation; (2) inhibit lipid peroxidation and so maintain cell membrane integrity—mainly those with high amounts of polyunsaturated fatty acids (PUFAs)—thus defending the cell against damage revealing a potent antioxidant power [88]; (3) increases immune response; (4) regulate platelet aggregation with its power to inhibit cyclooxygenase, reducing the formation of thromboxanes [85,89,90]; (5) inhibit the action of the protein kinase C [89]. Different tocotrienols were showed to participate in the inhibition of cell proliferation [91], delaying cancer [92] but not reversing a carcinogenic process that has already begun. Our results show elementary leaves of all cultivars to be richer in isomer α-T compared to leaf sprouts. Total tocopherol and α-T for all cultivars varied as Verdeal > Cobrançosa > Madural. α-T, the most compatible isomer with the human body’s, was showed to be the predominant form of vitamin E present for every variety studied in this work, representing 97.03% (Verdeal) > 96.01% (Madural) > 95.37% (Cobrançosa) for elementary leaves. For that, and in accordance with previous literature, it would be interesting to further study the OLE’s nutraceutical potential of Verdeal leaves to (1) fight against teratogenic effects; (2) improve osteoporosis [93] by inhibiting prostaglandins [94,95]; (3) heal periodontitis trough correction of redox status imbalance [96,97]; (4) increase the extent of collagen fiber deposition and reduced inflammatory response by nano-emulsion-encapsulating combinations with other therapeutic agents [98,99]; (5) keep a functional cell membrane by stabilizing PUFAs [99]; and (6) be valuable treatment for patients with allergic conditions [100,101]. On the other hand, Cobrançosa’s leaves, given their relative high content of α-T and TPC, appear a suitable substrate to obtain extracts with nutraceutical properties (1) to treat CVD, since α-T attenuates atheroma’s formation [102,103,104]; (2) to fix triglycerides plasma levels [54,105,106,107]; (3) to prevent the oxidation of LDL reducing cholesterol induced ER stress; (4) to prevent apoptosis by increasing proteasome activity in the hippocampus [108,109]; (5) to improve several chronic inflammatory skin diseases, such as vitiligo, psoriasis, atopic dermatitis, and acne [101]; (6) to treat respiratory infections with inflammatory processes such SARS-CoV-2; and (7) to boost anti-aging treatments. Plus, α-tocopherol is also known to be a safe food additive [110] and Cobrançosa’s leaf sprouts might be valuable in food preservation for their low water content associated with high TPC. Overall, vitamin E content of *O. europaea* L. *folium* might be an indicator that this substrate is a potential low-cost solution to produce vitamin E supplementation, either pure or reinforced with OA and/or polyphenols, depending on the therapeutic target. Nano-emulsion-encapsulation, with other therapeutic agents acting upon a synergetic anti-inflammatory capacity such as vitamin C, A, and other antioxidants, is a possible route for optimized efficiency in future supplementation. For that, we suggest future studies on vitamin E metabolism to understand the discrepancies observed in studies performed in vivo and in vitro using vitamin E as a supplement or nutrient.

### 3.4. Fatty Acids in O. europaea L. folium

Fatty acids’ profile showed a relative major presence of (PUFA) > (SFA) > (MUFA) for all samples, except for Cobrançosa’s leaf sprouts—SFA > PUFA > MUFA content. The PUFAs profile of all cultivars showed a predominance of omega-3 linolenic acid (ALA, C18:3n3,) in all leaves and leaf sprouts of the three cultivars. Both leaves and leaf sprouts showed a profile of ALA: Verdeal (40.42% and 37.35) > Madural (37.87% and 26.48%) > Cobrançosa (26.48% and 24.18%) (Table 4). Omega-6 linoleic acid (LA, C18:2n6c) displayed a 50% relative amount compared to ALA. Giving the latest studies, PUFAs, associated with other supplements or alone, might play key roles in the treatment of several health conditions. PUFAs are recommended as beneficial supplementation for children suffering from ailments resulting from a deficient ratio omega-3/omega-6, such as learning disorders, which are defined as an unexpected failure to acquire adequate abilities in reading, writing, or mathematic skills, not as a result of reduced intellectual ability, inadequate teaching, or social deprivation [111]. Attention and behavior issues associated with attention deficit hyperactivity disorder (ADHD) might improve with omega-3 supplements, given the fact that the plasma of children and teenagers have consistently shown lower levels of this FA [112]. Increased consumption of PUFAs are associated with reduced risk of multiple sclerosis (MS) [113,114], benefited appetite regulation and resolution of inflammation, relevant in anorexia nervosa (AN) [115], improved mood disorders by modulating synaptogenesis, and neurotrophic factor expression, neurogenesis, and neurotransmission [116]. We suggest OLE of Verdeal’s elementary leaves and leaf sprouts as a potential vegetable source of omegas supplementation to treat CNS-linked disorders such as poor cognition, depression, anxiety disorders, poor anger control, ADHD and accelerated neurodegeneration in the elderly, often associated to low levels of omega-3 [117]. Their higher content of α-T helps to preserve the PUFA structure, promoting longer effects in treating diseases related to low omega-3 bioavailability. Different treatments to address non-alcoholic steatohepatitis (NASH) include PUFA supplements, vitamin E, and insulin sensitizing agents with a focus on pioglitazone and statin agents. αT, a well-known antioxidant with a key role against lipid peroxidation in NASH pathogenesis [118], has achieved highest results when combined with ascorbic acid [119,120]. However, the evidence lacks considerable certainty concerning the effects of nutritional supplementation compared to no additional intervention on all clinical outcomes for patients with NAFLD [121]. For that, we suggest further studies for exploring the benefits of OLE using (1) Verdeal and Madural leaves as affordable sources of PUFA; (2) Verdeal’s elementary leaves for optimal omega-6/omega-3 ratio; (3) Cobrançosa’s elementary leaves as an affordable source of α-T; and (4) Cobrançosa’s leaf sprouts for high TPC and potential to mimic statins or pioglitazone.

The total content of MUFA’s is the lesser value in FAC for all the samples of each cultivar. Omega-9 OA (C18:1n9c) shows predominance compared to a vestigial presence of *cis*-11-eicosanoic acid (C20:1n9) for all samples. The values of OA were found to be slightly higher for all leaf sprouts samples: Cobrançosa’s (20.66% and 16.24%, respectively) > Madural > Verdeal. Omega-9 FA are synthesized endogenously in the human body but does not provide body’s full requirements; hence they are considered partially essential fatty acids. Studies indicate the beneficial effects of MUFA’s for CVD to be consistent with PUFAs, namely, for the relief systolic blood pressure (SBP) and diastolic blood pressure (DBP), improvement of high LDLc, and triglycerides (TAG) levels [122]. Furthermore, inclusion of OA (omega-9) in a diet—together with other nutrients known to be cardioprotective such as eicosapentaenoic acid (EPA), docosahexaenoic acid (DHA), folic acid, and vitamins A, B-6, D, and E—enhanced clinical values while reducing a variety of risk factors in men with peripheric vascular disease and intermittent claudication (PVD-IC), announcing itself as new evidence of the potential nutraceutical role for these compounds in the reduction of PVD-IC symptoms [123]. So, Cobrançosa, Verdeal, and Madural leaf sprouts can be proposed as potential economic substrates for extracting omega-9 FA to supplementation or co-adjuvating treatments used in hypertension and dyslipidemia, including hypercholesteremia. The SFA prolife was determined to follow the pattern for every cultivar: Palmitic acid (C16:0) > stearic acid (C18:0) > myristic acid (C14:0) > arichid acid (C20:0). Palmitic acid (C16:0) was identified as the major SFA component with very approximate values for all samples of leaves and leaf sprouts. Cobrançosa’s leaf sprouts display a higher composition in SFA (Table 4); however, this is counterbalance by a lower value in carbohydrates (Table 1) and higher antioxidant TPC (Table 2). Overall, it is safe to say that (1) OLE is rich of OA and LA (a MUFA and PUFA respectively); (2) OLE also consists of vitamin E and polyphenols which are powerful antioxidants, (3) OLE has been scientifically shown to protect the heart and blood vessels from plaques and ischemic injuries; (4) and so, OLE can be proposed as a nutraceutical to complement a balanced diet without detrimental risk for CVD despite the SFA content, especially for the Verdeal and Madural cultivars.

### 3.5. In Silico Integrator Essays

Our pilot in silico assay suggests that immune alterations correlated with several diseases such as cancer, autism, hypertension, anxiety, and depression might be well treated by a combination of food supplement intake and bio resonance treatments using frequential information recording from plants such as *O. europaea* and *Cydonia Oblonga* [100].

Recent studies suggest that *Olea europaea* can be used as a great and economic source of bioactive compounds and may have relevance in the prevention of diseases in which free radicals are implicated. As verified by four clinical cases (personal data), the electromagnetic frequency information (vibration in Hz waves) of a medicinal plant such as *Olea europaea*, resonating with the receptors of the extracellular matrix, might augment its bioactive effects [124] when traditionally gastrointestinally and metabolically absorbed. We tested this dual methodology in drug-resistant high blood pressure patients and the bio resonance treatment of *Olea europaea* “tea” electromagnetic frequencies prior to the real *Olea europaea* leaf tea drinking made the decrease of blood pressure more significant and stable in time. Thus, these preliminary studies, associated to descriptive and elementary analyses, can offer a rich research field to evaluate the potential effect of bioproducts under single or combined formulas.

## 4. Materials and Methods

### 4.1. Chemical Reagents

Absolute ethanol was obtained from Fisher Chemical (Loughborough, England). Methanol, gallic acid, Folin–Ciocalteu reagent, sodium carbonate (Na_2_CO_3_), boron trifluoride (BF3), and 1,4-dioxane were purchased from Sigma (St. Louis, MO, USA). Nitric acid (HNO_3_), hydrogen peroxide (H_2_O_2_), Kjeldahl tablets catalyst, sulphuric acid, boric acid, potassium hydroxide (KOH), anhydrous sodium sulfate (Na_2_SO_4_), and n-hexane (HPLC grade) were obtained from Merck (Darmstadt, Germany). Tocol (2-methyl-2-(4,8,12-trimethyl-tridecyl) chroman-6-ol) was obtained from Matreya Inc. (State College, PA, USA). Vitamin E standards were from Calbiochem (La Jolla, CA, USA). Fatty acid methyl ester standard mixture (FAME) Supelco 37 was obtained from Supelco (Bellefonte, PA, USA). Water was purified in a Milli-Q system (Millipore, Bedford, MA, USA).

### 4.2. Sample Collection

**Figure 2:**
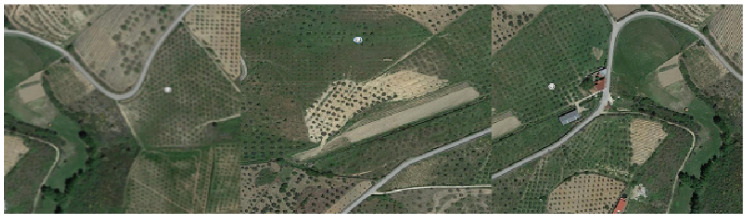
Photos of the harvesting local (aerial view obtained from Google maps according to the respective coordinates). Cultivar (from left to right): Madural, Verdeal, and Cobrançosa.

The olive samples from the olive grove of Valpaços in Santa Maria de Emeres village were collected in May. Two types of olive samples were considered: leaves and “*mamões*” (leaf sprouts), from three biological olive cultivars, namely, Madural, Verdeal e Cobrançosa. The coordinates in WGS84 for Madural is Lat: 41.540399, Long: −7.390831; for Verdeal Lat: 41.540463, Long: −7.396549; and for Cobrançosa Lat: 41.540290, Long: −7.394650 (Figure 2).

This area incorporated a total production territory of 8.9 ha, with 1.4 ha of Madural, 1.8 ha of Verdeal and 5.7 ha of Cobrançosa cultivars. Soil mobilization was conducted, and we planted a cover based on clover and others. We apply a calcium sulphate complex (Bordeaux mixture without copper), Sprintplus (Algae based), and mix organic matter with soil correctives according to the analysis. No pesticides or herbicides are used to treat the plants, and all products are properly certified for application in organic production or under biological conditions. The number of olive trees per unit of area, on average, is approximately 350 trees per 6 × 6 m^2^, but in the case of the terraces they are closer together. At the start of planting and production, we received sufficient financial support for a project—Ref. Young Farmer: PDR—Rural Development Program, 2012—to initiate the plantation of 3 ha of olive groves in 2018.

### 4.3. Sample Preparation

The preparation of olive leave samples followed the same procedure used for the analysis of Cydonia oblonga samples since the studied parameters were similar (Ferreira et al., 2022) [49]. Thus, olive leaf samples were grounded in a mill (Retsch Knife Mill GRINDOMIX GM 200, Retsch, Haan, Germany) before organic analysis. For inorganic evaluation, 0.2 g of dry leaf sample was digested using microwaves (MW) within a closed system at 170 °C using 1 mL of HNO_3_, 2 mL of H_2_O_2_, and 1 mL of H_2_O. After cooling, the vessel contents were transferred to volumetric flasks and the volume was made up of 25 mL of deionized water.

### 4.4. Moisture Content

Assessment of moisture content was determined in triplicate through infrared hygrometry readings at 105 °C using 1 ± 0.1 g of milled leaves sample (infrared balance, Scaltec model SMO01, Scaltec Instruments, Heiligenstadt, Germany).

### 4.5. Nutritional Analysis

Nutritional analysis was determined according to AOAC procedures (2019) [125]. Total protein content was determined by the Kjeldahl method (AOAC 920.153). The used nitrogen conversion factor was 6.25 (Tontisirin, 2003) [126]. Total fat content was determined by the Soxhlet method (AOAC 991.36). The ash content was determined by sample incineration at 500 °C (AOAC 920.153). Total carbohydrates were calculated by difference, according to Tontisirin (2003) [126].

### 4.6. Extraction and Quantification of Total Phenolic Content (TPC)

For the extraction of phenolic compounds, the mass/volume ratio was optimized using milled olive leaves and 80/20 methanol/water (*V/V*) as solvent. The mixture was agitated in a magnetic stirrer (MS-H-S10 magnetic stirrer, ChemLand, Usługowa, Stargard, Poland) at a constant temperature (40 °C) and agitation (600 rpm) for 1 h, according to Melo et al. (2021) [127]. The total phenolic content (TPC) was determined by a spectrophotometric method with the Folin–Ciocalteu reagent at room temperature using an absorbance reading of 765 nm in a microplate reader (BioTek Instruments, Synergy HT GENS5, Winooski, VT, USA) following Melo et al. (2021) [127]. Results are reported in g of gallic acid equivalents (GAE)/100 g of sample fresh weight.

### 4.7. Extraction of Lipid Fraction

According to Melo et al. (2021) [127], the lipid fraction extraction was accomplished by using absolute ethanol and n-hexane (HPLC grade) as solvents in constant agitation (Multi Reax EU, Heidolph, Schwabach, Germany). The final extract was used for vitamin E and fatty acid profile assessment.

#### 4.7.1. Vitamin E Profile

For vitamin E profile determination, the final extract (using tocol as an internal standard) was injected in an HPLC–DAD–FLD (high performance liquid chromatography with diode array detector and fluorescence detector) system (Jasco, Tokyo, Japan) equipped with an MD-4015 multiwavelength diode array detector (Jasco, Tokyo, Japan), and an FP-4025 fluorescence detector (Jasco, Tokyo, Japan) programmed for an excitation of λ = 290 nm, an emission of λ = 330 nm, a PV-4180 pump, an AS-4050 autosampler, and a normal phase Supelcosil TM LC-SI column (75 mm × 3.0 mm, 3.0 μm, Supelco, Bellefonte, PA, USA). The eluent was 1.2% 1,4-dioxane in n-hexane (*V*/*V*). The flow rate was 0.7 mL/min. The injection volume was 20 μL. Vitamin E isomers (α-tocopherol, β-tocopherol, γ-tocopherol, δ-tocopherol, α-tocotrienol, β-tocotrienol, γ-tocotrienol, and δ-tocotrienol) were identified based on their UV spectra (with maximum absorption around λ = 296 nm) and by comparison to the retention times of standards. Isomers were quantified based on the fluorescence signals and using calibration curves for each isomer plotted with individual commercial standards from Calbiochem (La Jolla, CA, USA) with purity ≥96%. Results are expressed in mg of each isomer/100 g of sample in fresh weight.

#### 4.7.2. Fatty Acids (FA) Profile

For FA profile determination, a transmethylation with KOH in methanol was performed on the extract to obtain methyl esters, according to ISO 12966-2:2017 [128]. The obtained extract was then injected in a GC-FID system (Shimadzu, Tokyo, Japan) equipped with an AOC-20i automatic sampler and a split/splitless auto injector (Shimadzu, Tokyo, Japan) at 250 °C, a flame ionization detector (Shimadzu, Tokyo, Japan) at 270 °C, and a CP-Sil 88 silica capillary column (50.0 m × 0.25 mm inner diameter and 0.20 μm film thickness, Varian, Middelburg, Netherlands). The carrier gas was helium. The injection volume was 1 μL. The used program was: 120 °C held for 5 min, 2 °C/min to 160 °C held for 2 min, and 2 °C/min to 220 °C held for 10 min. Identification was performed by comparing the retention times of fatty acids methyl esters to a standard mixture (FAME 37, Supelco, Bellefonte, PA, USA). The data were analyzed based on relative peak areas. Results are expressed as a relative percentage (%) of total FA.

### 4.8. In Silico Human Integrator: Test of Substance Effects

MORA Nova^®^ procedure is a diagnostically and therapeutic technique based on the findings of quantum physics and with historical roots in Dr. Voll’s bioresonance findings. This innovative method, developed in the mid-’70s, transfers the principle of waves and frequencies, from quantum physics to medical technology [124,129,130].

These in silico assays based on bioresonance with MORA Nova^®^ integrator were performed to analyze the potential therapeutic resonance of *O. europaea* regarding several physiological parameters. It is possible to predict its effects and properties attending neuroinflammatory, neurobehavioral, cardiovascular, pulmonary, arterial tension, metabolic and hormonal functions, osteo-degenerative, anti-ageing, and immunological defense disorders.

### 4.9. Statistical Analysis

Statistical analysis was performed using IBM SPSS Statistics (v. 26 for Windows, IBM Corp., Armonk, 241 NY, USA). The evaluation of statistical significance was determined by ANOVA and Tukey’s HSD to assess significant differences between samples at a 5% significance level.

## 5. Conclusions

From our study it is possible to conclude that Cobrançosa’s variety might be suggested as a safe and inexpensive prospect to extract polyphenols as pharmaceutical weapons for (1) improving hematologic disorders facing tall demands in public health such as anemia; (2) resolving thrombotic disorders; (3) improving hypercholesteremia. thus coming of age as a potential co-adjuvant or alternative to statins; (4) anti-hypertension supplements; (5) reducing hyperglycemia; (6) improving insulin sensitivity for treating conditions such as IR underlying T2DM, substituting or complementing metformin therapeutics; (7) possible natural synergetic treatment with other anti-cancerogenic; (8) an adjuvant treatment of prostatitis, preventing the transformation of hypertrophic cells into cancerous; (9) amelioration of several diseases associated with neuroinflammatory processes; (10) improving anti-aging processes dependent on ROS accumulation; (11) treating antipathogenic agents including bacteria, viruses, Trypanosoma, and leishmaniosis; and (12) promoting healthy probiotic gut flora. Moreover, the predominance of α-T allied to essential FA as PUFAs in Verdeal’s elementary leaves and sprout leaves is suggested as potential economic and high quality candidate in (1) supplementation for bone health mainly when combined with other such OA and polyphenols for post-menopausal women and patients with bone erosion; (2) fighting against periodontal signs via its anti-inflammatory properties; (3) the food industry as a safe preservative/additive; (4) supplementation for risk reducing CVD, CHD, or stroke; (5) management of dry eye syndrome; (6) preventing MS; (7) supplementation for ADHD; (8) pacifying treatment for mood disorders and other behavioral conditions such as depression, anxiety disorders, poor anger control, and AN; (9) accelerated neurodegeneration in elderly patients; and (10) preventing asthma and allergic diseases by decreasing omega-6 fatty acid and increasing omega-3. On the other hand, Verdeal’s elementary leaves, rich in OA, vitamin E, and polyphenols, can become a special nutraceutical for a prophylaxis against (1) heart and blood vessels plaque formation; (2) ischemic injuries associated with a balanced Mediterranean diet; and (3) to alleviate manage SARS-CoV-2 symptoms, specifically those related to exacerbated inflammatory processes [35,131,132]. On the other hand, given the fact that leaf sprouts often are discarded for overtaxing nutrients and energy from the central axis of the olive tree, and considering that leaf sprouts from every cultivar showed a considerable content in omega-9, these can also be suggested as a very accessible and valuable substrate for supplementation combined with EPA, DHA, folic acid, and other vitamins for improving symptoms of vascular diseases such PVD-IC, CVD, etc. Finally, Cobrançosa’s leaf sprouts combined with Verdeal’s elementary leaves for their lower moisture content, added up to their considerable amounts in TPC, FAC, and α-T, turns them into a powerful candidate for an optimal supplement against the anti-inflammatory diseases which currently burden public health. Overall, we would welcome to further study of the mechanisms acting behind these beneficial effects of olive leaves on neuroimmune and cardiovascular health, so that natural supplements could be designed to provide a safe complement or alternative, free of the deleterious side effects of classical pharmacological interventions currently employed in prevalent chronic diseases.

## Figures and Tables

**Table 1:** Nutritional analysis of different varieties of olive leaves.

	Cultivar	Madural		Verdeal		Cobrançosa	
Nutrient		Elementary Leaves	Leaf Sprouts (*mamões*)	Elementary Leaves	Leaf Sprouts (*mamões*)	Elementary Leaves	Leaf Sprouts (*mamões*)
Moisture		8.91 ± 0.09 ^a,b^	8.76 ± 0.18 ^a,b^	8.61 ± 0.12 ^a,b^	8.73 ± 0.01 ^a,b^	9.28 ± 0.42 ^b^	8.43 ± 0.11 ^a^
Ash		3.94 ± 0.02 ^a^	4.84 ± 0.12 ^b^	4.38 ± 0.35 ^a,b^	4.23 ± 0.03 ^a^	4.86 ± 0.09 ^b^	4.77 ± 0.04 ^b^
Total protein		7.44 ± 0.02 ^a^	9.04 ± 0.47 ^b^	8.85 ± 0.20 ^b^	10.72 ± 0.06 ^c^	8.74 ± 0.28 ^b^	11.66 ± 0.01 ^d^
Total fat		3.45 ± 0.01 ^c^	1.77 ± 0.22 ^a^	4.01 ± 0.14 ^d^	2.45 ± 0.04 ^b^	2.15 ± 0.22 ^a,b^	2.38 ± 0.17 ^b^
Total carbohydrates		76.25 ± 0.09 ^c^	75.60 ± 0.57 ^c^	74.15 ± 0.71 ^b^	73.87 ± 0.09 ^a,b^	74.96 ± 0.34 ^b,c^	72.75 ± 0.16 ^a^

Results are presented in percentage (%) in fresh weight (mean ± standard deviation, *n* = 3). Different lower case letters (a,b,c,d) denote significant differences (*p* < 0.05) by ANOVA, followed by Tukey’s post-hoc test (SPSS Statistics). Total carbohydrates were calculated by difference (%Total carbohydrates = 100% − [%Moisture + %Ash + %Total protein + %Total fat]).

**Table 2:** Total phenolic compounds of different varieties of olive leaves.

	Cultivar	Madural		Verdeal		Cobrançosa	
Polyphenol Content		Elementary Leaves	Leaf Sprouts (*mamões*)	Elementary Leaves	Leaf Sprouts (*mamões*)	Elementary Leaves	Leaf Sprouts (*mamões*)
TPC		2.31 ± 0.22 ^a^	3.59 ± 0.21 ^c,d^	2.47 ± 0.23 ^a^	2.87 ± 0.22 ^b^	3.37 ± 0.25 ^c^	3.78 ± 0.17 ^d^

Results reported in g of gallic acid equivalents (GAE)/100 g of leaves in fresh weight (mean ± standard deviation, *n* = 9). Different lower case letters (a,b,c,d) denote significant differences (*p* < 0.05) by ANOVA, followed by Tukey’s post-hoc test (SPSS Statistics). TPC—total phenolic compounds.

**Table 3:** Vitamin E profile of different varieties of olive leaves.

	Cultivar	Madural		Verdeal		Cobrançosa	
Isomer		Elementary Leaves	Leaf Sprouts (*mamões*)	Elementary Leaves	Leaf Sprouts (*mamões*)	Elementary Leaves	Leaf Sprouts (*mamões*)
α-T		11.55 ± 0.11 ^c^	3.50 ± 0.12 ^a^	20.97 ± 0.40 ^f^	13.75 ± 0.34 ^d^	17.92 ± 0.52 ^e^	8.07 ± 0.44 ^b^
β-T		0.21 ± 0.01 ^c^	0.15 ± 0.00 ^a^	0.32 ± 0.00 ^f^	0.26 ± 0.01 ^d^	0.30 ± 0.00 ^e^	0.19 ± 0.00 ^b^
γ-T		0.26 ± 0.01 ^a^	0.28 ± 0.01 ^a,b^	0.32 ± 0.00 ^b^	0.40 ± 0.02 ^c^	0.58 ± 0.01 ^d^	0.30 ± 0.01 ^a,b^
δ-T		ND	ND	ND	0.33 ± 0.02 ^a^	ND	ND
Total		12.03 ± 0.11 ^c^	3.94 ± 0.12 ^a^	21.61 ± 0.40 ^f^	14.74 ± 0.35 ^d^	18.79 ± 0.54 ^e^	8.56 ± 0.45 ^b^

Results are presented in mg/100 g of leaves in fresh weight (mean ± standard deviation, *n* = 3). Different lower case letters (a,b,c,d) denote significant differences (*p* < 0.05) by ANOVA, followed by Tukey’s post-hoc test (SPSS Statistics). T—tocopherol, ND—not detected.

**Table 4:** Fatty acids profile of different varieties of olive leaves.

	Cultivar	Madural		Verdeal		Cobrançosa	
Fatty Acid		Elementary Leaves	Leaf Sprouts (*mamões*)	Elementary Leaves	Leaf Sprouts (*mamões*)	Elementary Leaves	Leaf Sprouts (*mamões*)
C14:0		2.83 ± 0.03 ^b^	2.44 ± 0.24 ^b^	2.36 ± 0.35 ^b^	0.92 ± 0.08 ^a^	2.72 ± 0.05 ^b^	2.35 ± 0.13 ^b^
C16:0		24.73 ± 0.18 ^a,b^	26.54 ± 0.39 ^c^	25.07 ± 0.54 ^b^	23.68 ± 0.41 ^a^	25.61 ± 0.18 ^b,c^	28.60 ± 0.52 ^d^
C16:1		0.65 ± 0.02 ^a,b^	0.70 ± 0.05 ^a,b^	0.67 ± 0.03 ^a,b^	0.61 ± 0.03 ^a^	0.76 ± 0.03 ^b,c^	0.85 ± 0.08 ^d^
C17:0		0.32 ± 0.02 ^a^	0.42 ± 0.06 ^a^	0.34 ± 0.01 ^a^	0.33 ± 0.04 ^a^	0.39 ± 0.02 ^a^	0.36 ± 0.06 ^a^
C18:0		3.74 ± 0.02 ^b^	5.56 ± 0.43 ^c^	3.39 ± 0.29 ^a,b^	3.29 ± 0.08 ^a,b^	2.88 ± 0.12 ^a^	4.95 ± 0.48 ^c^
C18:1n9c		13.53 ± 0.50 ^a^	16.72 ± 2.27 ^a,b^	12.59 ± 1.60 ^a^	16.44 ± 0.38 ^a,b^	16.24 ± 1.44 ^a,b^	20.66 ± 2.61 ^b^
C18:2n6c		11.78 ± 0.72 ^a^	16.11 ± 0.82 ^c^	12.06 ± 0.30 ^a^	14.58 ± 0.14 ^b^	13.38 ± 0.27 ^b^	14.17 ± 0.12 ^b^
C18:3n3		37.87 ± 0.55 ^b,c^	26.48 ± 1.36 ^a^	40.42 ± 2.07 ^d^	37.35 ± 0.33 ^b,c^	34.45 ± 1.27 ^b^	24.18 ± 2.33 ^a^
C20:0		2.15 ± 0.06 ^b^	2.31 ± 0.30 ^b^	1.35 ± 0.03 ^a^	1.17 ± 0.05 ^a^	1.45 ± 0.05 ^a^	1.40 ± 0.12 ^a^
C20:1n9		0.23 ± 0.02 ^a^	0.48 ± 0.04 ^d^	0.27 ± 0.02 ^a,b^	0.23 ± 0.01 ^a^	0.40 ± 0.03 ^c^	0.33 ± 0.01 ^b^
C21:0		0.55 ± 0.04 ^c^	0.62 ± 0.03 ^c^	0.26 ± 0.03 ^a,b^	0.19 ± 0.03 ^a^	0.29 ± 0.02 ^b^	0.31 ± 0.03 ^b^
C22:0		1.14 ± 0.03 ^d^	ND	0.80 ± 0.04 ^b^	0.91 ± 0.10 ^b,c^	0.95 ± 0.01 ^c^	0.53 ± 0.03 ^a^
C23:0		0.46 ± 0.03 ^a^	1.61 ± 0.19 ^c^	0.41 ± 0.04 ^a^	0.28 ± 0.02 ^a^	0.50 ± 0.02 ^a^	1.31 ± 0.03 ^b^
∑SFA		35.93 ± 0.26 ^c^	39.51 ± 0.07 ^d^	33.98 ± 1.04 ^b^	30.78 ± 0.26 ^a^	34.78 ± 0.17 ^b,c^	39.80 ± 0.37 ^d^
∑MUFA		14.41 ± 0.51 ^a^	17.91 ± 2.21 ^a,b^	13.54 ± 1.63 ^a^	17.28 ± 0.40 ^a,b^	17.39 ± 1.44 ^a,b^	21.84 ± 2.67 ^b^
∑PUFA		49.65 ± 0.35 ^b^	42.59 ± 2.18 ^a^	52.48 ± 2.36 ^b^	51.94 ± 0.41 ^b^	47.83 ± 1.29 ^b^	38.36 ± 2.35 ^a^

Results presented as relative percentage (%) of total fatty acids (mean ± standard deviation, *n* = 3). Different lower case letters (a,b,c,d) denote significant differences (*p* < 0.05) by ANOVA, followed by Tukey’s post-hoc test (SPSS Statistics). C14:0—myristic acid, C16:0—palmitic acid, C16:1—palmitoleic acid, C17:0—margaric acid, C18:0—stearic acid, C18:1n9c—oleic acid, C18:2n6c—linoleic acid, C18:3n3—linolenic acid, C20:0—arachidic acid, C20:1n9—cis-11-eicosanoic acid, C22:0—behenic acid, C23:0—tricosanoic acid, C24:0—lignoceric acid, SFA—saturated fatty acids, MUFA—monounsaturated fatty acids, PUFA—polyunsaturated fatty acids, ND—not detected.

## Data Availability

Not applicable.
